# Up and down from North to South: Latitudinal Distribution of Flea Beetle Genera in Continental Africa (Coleoptera, Chrysomelidae, Galerucinae, Alticini)

**DOI:** 10.3390/insects14040394

**Published:** 2023-04-18

**Authors:** Maurizio Biondi, Paola D’Alessandro, Mattia Iannella

**Affiliations:** Department of Life, Health & Environmental Sciences, University of L’Aquila, Via Vetoio—Coppito, 67100 L’Aquila, Italy; maurizio.biondi@univaq.it (M.B.); mattia.iannella@univaq.it (M.I.)

**Keywords:** Africa, Chrysomelidae, latitudinal distribution, phytophagous insects

## Abstract

**Simple Summary:**

Biodiversity is not evenly distributed on Earth. For phytophagous insects, we could expect increasing taxonomic richness from temperate to tropical latitudes, where plant diversity is high. In this paper, we explored the variation in the number of genera in one of the most widespread groups of phytophagous insects, flea beetles, from north to south on the African continent. We found that the number of genera depends on the number of vegetation types, the kind of vegetation, and some specific bioclimatic variables, leading to an up-and-down trend in taxonomic richness from north to south.

**Abstract:**

The distribution of global biodiversity can be investigated based on comprehensive datasets and many methods to process them. The taxonomic diversity of phytophagous insects is typically linked to plant diversity, which increases from temperate to tropical latitudes. In this paper, we explored the latitudinal distribution of the flea beetle genera (Coleoptera, Chrysomelidae, Galerucinae, Alticini) on the African continent. We divided the area into latitudinal belts and looked for possible correlations with the number and types of vegetational divisions, the area of each belt, and the bioclimatic variables. The number of flea beetle genera is related to the number and types of vegetation divisions rather than the area of each belt. Some bioclimatic variables are highly related to the number of genera, which is higher within those belts where climate factors limit the oscillation of temperature over the year and favor high precipitations, especially in the warmest months. These biotic and abiotic factors lead to a two-peak trend in the taxonomic richness of flea beetle genera from north to south. Genera endemic to restricted areas are linked to the presence of high mountain systems and increase the taxonomic richness of the belt they belong to.

## 1. Introduction

Biodiversity is not evenly distributed on Earth [1]. The recent emergence of comprehensive global datasets on species occurrences, the availability of genetic datasets, and new methods for processing them have facilitated global analyses of biodiversity distribution and investigations of the factors shaping them [2,3,4]. Major studies have focused on vertebrates and plants as model systems that are generally used to explore and test the latitudinal diversity gradient, which is recognized as the main pattern in the distribution of life [5,6,7,8,9,10]. Invertebrate distribution, instead, has been comparatively poorly investigated, arguably due to the data deficiency for most taxa at the global or continental scales. Regarding terrestrial invertebrates, studies were conducted on specific insect groups [11,12,13,14,15,16,17]. However, the occurrence of a biodiversity gradient has not been documented for most taxa.

This paper explored the latitudinal distribution of a tribe of leaf beetles, Alticini, at the continental scale, in Africa. With over 40,000 species worldwide, leaf beetles (Coleoptera Chrysomelidae) are one of the most abundant families of phytophagous insects and are widespread in the terrestrial habitats of all continents. Alticini, defined as “flea beetles” due to their ability to jump, are the largest and most diverse tribe of leaf beetles in the subfamily Galerucinae, which also includes pests and alien species [18,19,20,21,22,23]. They occur worldwide, except in the polar regions, with 601 genera and about 10,000 species [24]. Most are highly specialized phytophagous insects that are primarily associated with herbaceous plants and, to a lesser extent, shrubs and trees. The adult and larval stages mainly feed on the stems, leaves, or roots of most higher plant families in different environments but rarely feed on the flowers **[25,26]**. Alticini can be a reliable model to explore phytophagous insects’ distributional patterns thanks to their wide distribution, taxonomic richness at the species and genus levels, and various trophic strategies. [27]. The African flea beetle fauna has been the object of extensive studies over the last 30 years (see references for occurrence data in the Section 2) despite still needing deep investigations for some areas. We investigated the variation in the number of genera and the possible correlation of this number with the sampling area, vegetation type, and bioclimatic variables.

## 2. Materials and Methods

### 2.1. Study Area, Dataset, Vegetation Formations, and Bioclimatic Variables

The study area consisted of continental Africa. Madagascar was excluded from our analyses because the knowledge of the flea beetle fauna in this region is very poor and not comparable with that of the African continent [28,29,30,31,32]. The African continent was divided into 15 latitudinal belts of 5°, a spatial resolution that reduced potential biases due to the possible incompleteness of the occurrence data. The area of each belt was calculated in ArcGIS Pro 3.01 [33], projecting the spatial data in the WGS 1984 Sinusoidal Africa (EPSG: 102,011) while concurrently taking into account the geodesy in the calculation.

Occurrence data of flea beetles were obtained from checked literature [34,35,36,37,38,39,40,41,42,43,44,45,46] and integrated with unpublished data from entomological collections preserved in numerous depositories worldwide (Biondi, unpublished data). We conducted the study at the genus level to avoid biases in the number of taxa in each belt. Species-level data may be affected by taxonomic uncertainties and potential misidentifications [47], while genera are comparatively stable taxonomic entities, especially in areas where faunal knowledge is still very partial. Although some authors have recently attributed the genera *Hespera* and *Luperomorpha* to the “Galerucini *incerta sedis*” group [24,48], in this work, they were considered Alticini, pending a definite taxonomic collocation.

The taxonomic richness of phytophagous insects is typically linked to vegetation features. Thus, to assess possible correlations of the number of genera in each latitudinal belt with vegetation types, we gathered spatial information on a raster map of the terrestrial ecosystems of Africa where vegetation formations were classified hierarchically (i.e., class, subclass, formation, division, and macro-group) [49].

Because abiotic factors may have independently affected the taxonomic richness, we evaluated possible correlations of the number of genera with some bioclimatic variables. We used the 19 temperature- and precipitation-related variables that are available on the Worldclim 2.1 online repository at a 2.5 min spatial resolution [50].

### 2.2. Statistical and Spatial Analyses

The correlations of the number of genera in each latitudinal belt with the sampling area, vegetation type, and bioclimatic variables were evaluated using the Pearson correlation coefficient (*r*), which was calculated using the statistical package NCSS version 11 for Windows [51].

Geostatistical analyses were conducted using ArcGis Pro 3.01 [33]. Specifically, 5° latitudinal belts were generated, spanning from 40–35° N to 30–35° S, thus encompassing the whole of Africa. Then, we intersected those belts with Africa’s boundaries, obtaining specific latitudinal belts for this continent. Then, we used each as a crop mask to extract information from the African vegetation types raster dataset [49], subsequently calculating the area of each vegetation type per latitudinal belt.

A cluster analysis was performed to highlight the degree of association between the latitudinal belts considered here and flea beetle genera. The web tool ClustVis [52] was used to generate a binary heatmap using hierarchical clustering, applying Euclidean distance and Ward linkage for areas and genera. The analysis was returned as single output clusters of genera based on the similarity of their distributions and clusters of areas based on their faunistic similarity. The following areas were introduced in the analysis to also take into account the occurrence of genera outside of the African continent: the Arabian Peninsula (AP), the Australian region (AUR), Madagascar (MAD), the Nearctic region (NAR), the Neotropical region (NTR), the Oriental region (ORR), and the Palearctic region (PAR).

## 3. Results

At the current state of knowledge, 96 genera of Chrysomelidae Alticini occur on the African continent (Table 1), of which 78.12% (74) are only present in sub-Saharan Africa and 11.46% (11) are only present in Mediterranean Africa, while 10.42% (10) are widespread on the entire continent. Regarding the 74 genera of the sub-Saharan component, 58.66% (44) are strictly endemic to the African continent, 10.67% (8) are shared with only Madagascar, and 4.00% (3) are shared with only the Arabian Peninsula. The other 26.67% of the sub-Saharan genera (20) have the widest distributions and are mainly extended to the Oriental (18) and Palearctic (15) regions. None of the 11 flea beetle genera present in only Mediterranean Africa are endemic. All of them are widespread in Europe; of these, 36.36% (4) also occur in the Nearctic region, and 27.27% (3) are also present in the Oriental region. Finally, the pan-African component includes genera that are also widely distributed in other zoogeographical regions, except for the genus *Angulaphthona*, which is endemic to the African continent, with a short extension into the Arabian Peninsula.

**Figure 1:**
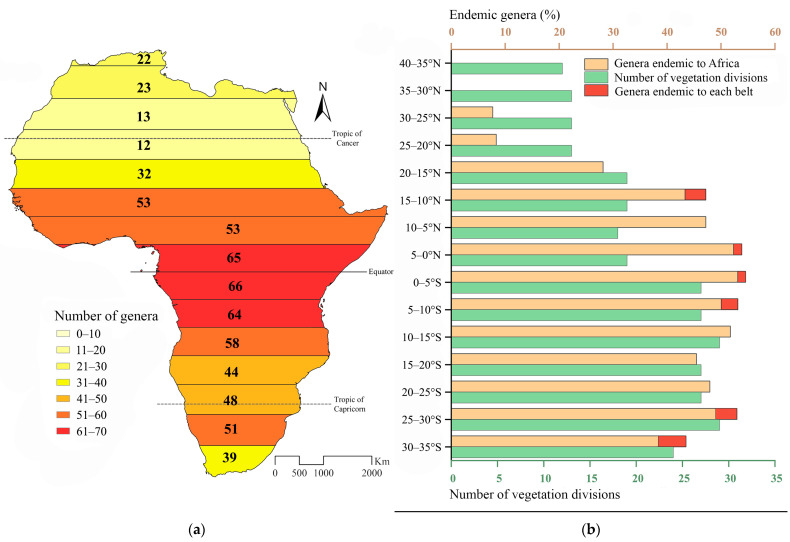
(**a**) The number of flea beetle genera within the 15 latitudinal belts that were considered. (**b**) The percentage of African endemic genera (orange, with the fraction of endemic genera exclusive to the belt in red) and the number of vegetation divisions [49] (green) within the 15 latitudinal belts that were considered.

The latitudinal distribution of the number of flea beetle genera in Africa has an approximately sinusoidal trend (Figure 1a), with a relative maximum in Mediterranean Africa (40–30° N), a minimum in correspondence with the Sahara Desert (30–20° N), and an increase with an absolute maximum in the equatorial belts (5° N–5° S). South of the equator, the genus richness decreases in the latitudinal belts that include the Namib and Kalahari deserts and increases significantly in the more southern temperate belts.

The African endemic component is composed of 44 genera that are exclusively present in sub-Saharan Africa, with limited extensions into the Saharan area (Figure 1b). Considered alone, it shows a latitudinal trend in the distribution similar to that of the entire flea beetle fauna (Figure 1a,b). It starts from 30° N, with an absolute maximum in the equatorial belts (5–0° N and 0–5° S, with 53.85% and 54.55% of the endemic component, respectively) and a relative maximum in the temperate belt (25–30° S and 30–35° S, with 52.49% and 43.49% of the endemic component, respectively).

**Figure 2:**
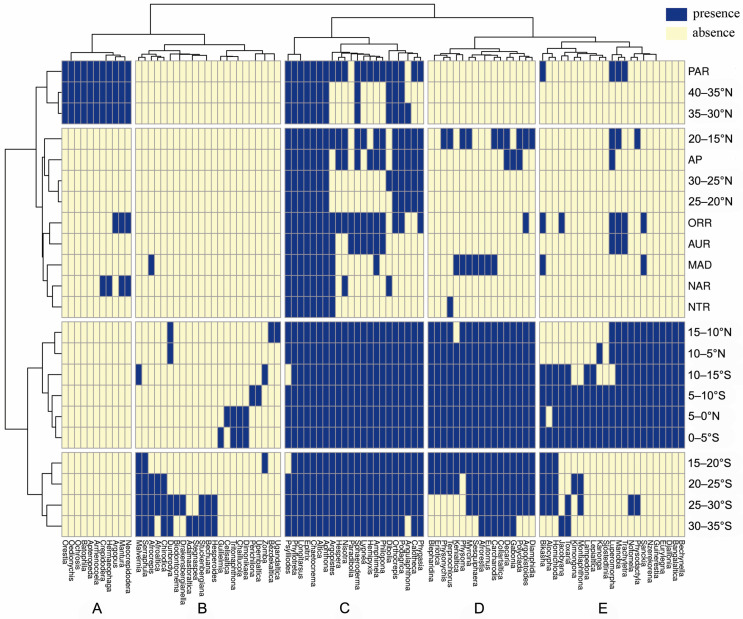
Binary heatmap from the hierarchical clustering with the genera and areas included in the analysis. AP: Arabian Peninsula, AUR: Australian region, MAD: Madagascar, NAR: Nearctic region, NTR: Neotropical region, ORR: Oriental region, PAR: Palearctic region.

The binary heatmap obtained from the cluster analysis is reported in Figure 2, where flea beetle genera are clustered based on the similarity of their distributions and the areas are clustered based on their faunistic similarity. The 11 genera that are only present in Mediterranean Africa, north of the Sahara Desert, are gathered in block A. They are all shared with the Western Palearctic region, and some are also shared with the Nearctic region (*Crepidodera* and *Hermaeophaga*), the Oriental region (*Argopus*), or with both (*Mantura* and *Neocrepidodera*). Cluster B includes most sub-Saharan endemic genera, particularly those distributed S of latitude 15–10° N; only *Afrocrepis* is also present in Madagascar. It includes *Adamastoraltica*, *Biodontocnema*, *Chirodica*, *Drakensbergianella*, and *Stegnaspea*, which are endemic to southern Africa, and *Celisaltica*, *Chaillucola*, *Dimonikaea*, *Guilielmia*, *Perichilona*, *Tritonaphthona,* and *Upembaltica*, which are more typically Central African. Block C comprises genera with broader distributions on the African continent that are present in other zoogeographical regions, mainly the Palearctic, Oriental, and Australian regions. The analysis also included the genus *Angulaphthona* in this cluster. It is widespread from north to south on the African continent, with a short extension into the Arabian Peninsula. Cluster D mainly groups genera endemic to the Afrotropical region, which are more widespread than those in cluster B and are generally also present in Madagascar and/or the Arabian Peninsula. Finally, block E consists of genera that are distributed mainly in the northernmost belts of sub-Saharan Africa. They are endemic to this area (e.g., *Bangalaltica*, *Bechynella*, *Djallonia*, *Eurylegna*, *Guinerestia*, and *Nzerekorena*) or are shared with other regions, especially the Australian and Oriental regions and, to a lesser extent, the Palearctic region.

**Figure 3:**
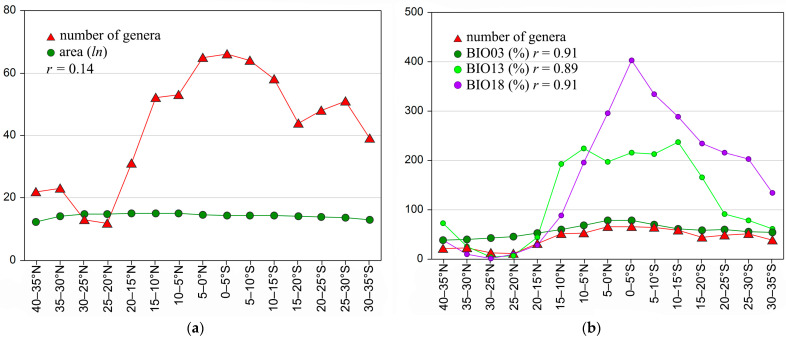
The number of flea beetle genera of the 15 latitudinal belts and (**a**) the area (*ln*) of each belt and (**b**) the values of the three bioclimatic variables [50] with the strongest positive correlations with the number of genera. *r*: Pearson correlation coefficient.

**Figure 4:**
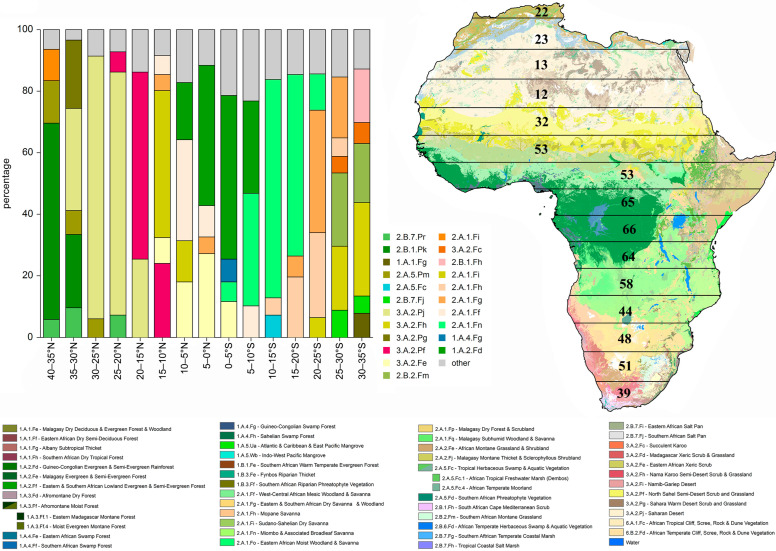
Main vegetational divisions [49] within the 15 latitudinal belts (**left**). Vegetation map and number of flea beetle genera in each belt (**right**).

The number of genera in the 15 latitudinal belts is uncorrelated with the area (*ln*) of each belt (*r* = 0.14) (Figure 3a and Table 2). It is instead significantly correlated with the number of vegetational divisions (*r* = 0.74) (Figure 1a,b (green bars), Figure 4, and Table 2). The number of genera also shows strong positive correlations with some of the bioclimatic variables considered here: BIO3 (isothermality: *r* = 0.91), BIO13 (precipitation of the wettest month: *r* = 0.89), and BIO18 (precipitation of the warmest quarter: *r* = 0.91) (Figure 3b and Table 3).

Figure 4 shows the main vegetational divisions within the 15 latitudinal belts and a vegetation division map with the number of flea beetle genera in each belt. The northernmost latitudinal belt, including Mediterranean Africa (40–35° N), hosts significant taxonomic richness (22 genera), despite occupying a relatively small area (219.406 km^2^). It is mainly characterized by the vegetational division “Northern African Mediterranean Scrub” (2.B.1.Pk), which occupies 63.74% of its area. Moving southward, belts 30–25° N and 25–20° N have an evident increase in the surface occupied by the “Saharan Desert” (3.A.2.Fh) (85.42% and 79.06%, respectively), which is accompanied by a significant decrease in the number of flea beetle genera (13 and 12, respectively), despite the large areas covered by these belts (2,567,593 km^2^ and 2,990,429 km^2^, respectively). A marked increase in the number of genera is observed approaching the equator. In the belt 20–15° N, a substantial increase in the number of genera (32) is combined with the extension of the “North Sahel Semi-Desert Scrub & Grassland” (3.A.2.Pf) (60.68%) and the contraction of the Saharan Desert (25.44%). The two southernmost pre-equatorial belts (15–10° N and 10–5° N) show further growth in the number of genera (53 in both cases) and the extension of savannah vegetation (“Sudano-Sahelian Dry Savanna” (2.A.1.Fi): 47.72%; “West-Central African Mesic Woodland & Savanna” (2.A.1.Ff): 32.84%), and, to a lesser extent, forestry (“Guineo-Congolian Evergreen & Semi-Evergreen Rainforest” (1 .A.2.Fd): 18.45%). The strictly equatorial latitudinal belts (5–0° N and 0–5° S) host the highest numbers of flea beetle genera (65 and 66, respectively). They are primarily occupied by the “Guineo-Congolian Evergreen & Semi-Evergreen Rainforest” (1.A.2.Fd), with 45.57% of the area in the northern belt (5–0° N) and 53.03% of the area in the southern one (0–5° S). In austral Africa, the two latitudinal belts 5–10° S and 10–15° S (north of the Tropic of Capricorn) host similar taxonomic richness (64 and 58 genera, respectively). The subsequent four temperate belts from 15° S to 35° S have comparatively smaller numbers of genera (44, 48, 51, and 39, respectively), especially in correspondence with the Namib and Kalahari deserts (15–25° S). Still, these numbers are decidedly higher than in the analogous belts of boreal Africa. From the vegetational point of view, the two northernmost belts (15–20° S and 20–25° S) are mainly characterized by savannahs (“Sudano-Sahelian Dry Savanna” (2.A.1.Fi); “Mopane Savanna” (2.A.1.Fh); and “Miombo & Associated Broadleaf Savanna” (2.A.1.Fn)), with total coverage values of 85.31% and 79.38%, respectively. The two southernmost belts (25–30° S and 30–35° S) are the only ones characterized by the presence of karoo vegetation (“Nama Karoo Semi-Desert Scrub & Grassland” (3.A.2.Fh) and “Succulent Karoo” (3.A.2.Fc), 26.13% and 37.40%, respectively), and high-altitude pasture (“Southern African Montane Grassland” (2.B.2.Fm)) (23.76% and 19.15%, respectively). Despite their small areas (915.547 km^2^ and 478.961 km^2^, respectively), they have proportionally high numbers of genera (51 and 39, respectively). The 30–35° S belt is the only one hosting Mediterranean scrub (“South African Cape Mediterranean Scrub” (2.B.1.Fh)), which occupies 17.26% of its surface area.

## 4. Discussion

We expected that the richness of phytophagous insects is linked to plant diversity, which increases from temperate to tropical latitudes [53,54]. Our results also showed this general latitudinal trend for the flea beetle genera in continental Africa (Figure 1a,b). More specifically, the number of genera is significantly correlated with the number of vegetational divisions [49], rather than the area of each latitudinal belt. Different vegetation types necessarily produce different ecological gradients that can favor biological diversification [55]. In addition, ecotones between vegetation types can favor edge effects, including increases in taxonomic richness [56,57].

However, single vegetation types also play a significant role in shaping taxonomic richness. In some cases, the difference in the number of genera is related to the different extensions or contractions of a specific vegetational type, rather than the number of vegetational divisions. For example, the increase in the taxonomic richness in the belt 20–15° N compared to the belt 25–20° N is combined with the extension of scrub vegetation and grasslands and the contraction of the Saharan Desert; the increase in the belt 5–0° N compared to the belt 10–5° N is combined with the extension of the evergreen rainforests and the contraction of the dry and mesic savannah; the decrease in the belt 15–20° S compared to the belt 10–15° S is mainly combined with the extension of the drier mopane savannah and the contraction of the wetter miombo and broadleaf savannah.

Both insects and plants also respond to abiotic factors that may have played independent roles in shaping current biodiversity patterns [58,59,60]. In our analysis, taxonomic richness appears to be higher where seasonality is absent and temperature oscillations over the year are comparable to the day-to-night temperature oscillations (the day-to-night temperature oscillations represent about 70–75% of the summer-to-winter variations); this occurs mainly in the equatorial area. Moreover, high mean values of precipitation in the wettest month (BIO13 ≥ 200 mm) and warmest quarter (BIO18 ≥ 300 mm) seem to favor the presence of a higher number of flea beetle genera in Africa.

These factors lead to a two-peak trend in the taxonomic richness of flea beetle genera from north to south in continental Africa.

Regarding the endemic component, most of the 44 genera occur in more than one latitudinal belt. Therefore, they are a representative subset of the whole ensemble of the sub-Saharan flea beetle fauna. They are subjected to the same factors affecting the taxonomic richness and thus show a similar latitudinal trend in the number of genera. The presence of genera exclusively associated with a single belt is mainly related to the occurrence of mountain systems, such as *Celisaltica* in the Ruwenzori Massif (Uganda), *Perichilona* in the Iringa region (Tanzania), *Upembaltica* in the Katanga region (Democratic Republic of Congo), and *Drakensbergianella* in the Drakensberg Mountains (Democratic Republic of South Africa). This is not surprising, considering the acknowledged role of tropical mountains as “cradles” and/or “museums” of biodiversity [10,11,61,62,63]. Indeed, although other mountains, such as Mount Kenya, Mount Aberdare, and Mount Kilimanjaro, lack endemic flea beetle genera, they nonetheless host several endemicities at the species level.

## Figures and Tables

**Table 1:** Flea beetle genera on the African continent, species number for each genus, and distribution. AP: Arabian Peninsula, AFR: African continent, AUR: Australian region, MAD: Madagascar, MAF: Mediterranean Africa, NAR: Nearctic region, NTR: Neotropical region, ORR: Oriental region, PAR: Palearctic region, SSA: sub-Saharan Africa.

Genera	No. of Species in Continental Africa	Distribution
*Adamastoraltica* Biondi, Iannella and D’Alessandro, 2020	1	SSA
*Afroaltica* Biondi and D’Alessandro, 2007	2	SSA
*Afrocrepis* Bechyné, 1954	3	SSA-MAD
*Afrorestia* Bechyné, 1959	≈20	SSA-MAD
*Alocypha* Weise, 1911	1	SSA
*Altica* Geoffroy, 1762	>30	World
*Amphimela* Chapuis, 1875	>30	SSA-AP-MAD-PAR-ORR-AUR
*Angulaphthona* Bechyné, 1960	7	AFR-AP
*Aphthona* Chevrolat, 1836	>30	World
*Apteropeda* Motschulsky, 1860	1	MAF-PAR
*Argopistes* Motschulsky, 1860	≈10	MAF-PAR
*Argopistoides* Jacoby, 1892	4	SSA-ORR
*Argopus* Fischer von Waldheim, 1824	1	MAF-PAR-ORR
*Arrhenocoela* Foudras, 1861	1	MAF-PAR
*Bangalaltica* Bechyné, 1960	1	SSA
*Batophila* Foudras, 1860	1	MAF-PAR
*Bechuana* Scherer, 1970	2	SSA
*Bechynella* Biondi and D’Alessandro, 2010	3	SSA
*Bezdekaltica* Döberl, 2012	1	SSA
*Bikasha* Maulik, 1931	≈6	SSA-MAD-PAR-ORR
*Biodontocnema* Biondi, 2000	1	SSA
*Blepharidina* Bechyné, 1968	>30	SSA
*Calotheca* Heyden, 1887	>30	SSA-AP-PAR
*Carcharodis* Weise, 1910	7	SSA-MAD
*Celisaltica* Biondi, 2001	1	SSA
*Chaetocnema* Stephens, 1831	>30	World
*Chaillucola* Bechyné, 1968	1	SSA
*Chirodica* Germar, 1834	8	SSA
*Collartaltica* Bechyné, 1959	6	SSA
*Crepidodera* Chevrolat, 1836	3	MAF-PAR-NAR
*Decaria* Weise, 1895	≈20	SSA-AP
*Diamphidia* Gerstaecker, 1855	17	SSA
*Dibolia* Latreille, 1829	≈20	AFR-PAR-NAR
*Dimonikaea* Bechyné, 1968	1	SSA
*Djallonia* Bechyné, 1955	1	SSA
*Drakensbergianella* Biondi and D’Alessandro, 2003	1	SSA
*Dunbrodya* Jacoby, 1906	2	SSA
*Epitrix* Foudras, 1860	≈12	World
*Eriotica* Harold, 1877	7	SSA
*Eurylegna* Weise, 1910	6	SSA
*Eutornus* Clark, 1860	≈7	SSA-MAD
*Gabonia* Jacoby, 1893	>30	SSA-AP
*Guilielmia* Weise, 1924	2	SSA
*Guinerestia* Scherer, 1959	3	SSA
*Hemipyxis* Chevrolat, 1836	>30	SSA-AP-PAR-ORR-AUR
*Hermaeophaga* Foudras, 1860	1	MAF-PAR-NAR
*Hespera* Weise, 1889	>30	SSA-AP-PAR-ORR
*Hesperoides* Biondi, 2017	1	SSA
*Homichloda* Weise, 1902	3	SSA
*Jacobyana* Maulik, 1926	3	SSA-ORR
*Kanonga* Bechyné, 1960	1	SSA
*Kenialtica* Bechyné, 1960	7	SSA-MAD
*Kimongona* Bechyné, 1959	3	SSA
*Lampedona* Weise, 1907	3	SSA
*Lepialtica* Scherer, 1962	4	SSA
*Longitarsus* Berthold, 1827	>30	World
*Luperomorpha* Weise, 1887	2	SSA-AP-PAR-ORR-AUR
*Lypnea* Baly, 1876	≈10	SSA-PAR-ORR-AUR
*Malvernia* Jacoby, 1899	2	SSA
*Manobia* Jacoby, 1885	≈15	SSA-PAR-ORR-AUR
*Mantura* Stephens, 1831	4	MAF-PAR-NAR-ORR
*Montiaphthona* Scherer, 1961	6	SSA
*Myrcina* Chapuis, 1875	≈16	SSA-MAD
*Neocrepidodera* Heikertinger, 1911	5	MAF-PAR-NAR-ORR
*Nisotra* Baly, 1864	>30	SSA-AP-PAR-ORR-AUR
*Notomela* Jacoby, 1899	3	SSA
*Nzerekorena* Bechyné, 1955	9	SSA
*Ochrosis* Foudras, 1861	1	MAF-PAR
*Oedionychis* Latreille, 1829	2	MAF-PAR
*Orestia* Chevrolat, 1836	3	MAF-PAR
*Orthocrepis* Weise, 1888	>30	AFR-AP-PAR-ORR
*Paradibolia* Baly, 1875	3	SSA-ORR-AUR
*Perichilona* Weise, 1919	2	SSA
*Philopona* Weise, 1903	≈20	SSA-AP-PAR-ORR-AUR
*Phygasia* Chevrolat, 1836	>30	SSA-AP-PAR-ORR
*Phyllotreta* Chevrolat, 1836	>30	World
*Physodactyla* Chapuis, 1875	6	SSA
*Physoma* Clark, 1863	2	SSA-MAD
*Physonychis* Clark, 1860	>30	SSA
*Podagrica* Chevrolat, 1836	>30	SSA-AP-PAR-ORR
*Polyclada* Chevrolat, 1836	16	SSA-AP
*Psylliodes* Berthold, 1827	9	World
*Sanckia* Duvivier, 1891	4	SSA-MAD-ORR
*Serraphula* Jacoby, 1897	19	SSA
*Sesquiphaera* Bechyné, 1958	≈10	SSA-MAD
*Sjostedtinia* Weise, 1910	2	SSA
*Sphaeroderma* Stephens, 1831	>30	SSA-AP-PAR-ORR-AUR
*Stegnaspea* Baly, 1877	6	SSA
*Stuckenbergiana* Scherer, 1963	1	SSA
*Terpnochlorus* Fairmaire, 1904	2	SSA-NTR
*Toxaria* Weise, 1903	5	SSA
*Trachytetra* Sharp, 1886	5	SSA-PAR-ORR-AUR
*Tritonaphthona* Bechyné, 1960	1	SSA
*Ugandaltica* D’Alessandro and Biondi, 2018	1	SSA
*Upembaltica* Bechyné, 1960	1	SSA
*Zomba* Bryant, 1922	1	SSA

**Table 2:** Metadata for the 15 latitudinal belts that were considered. Vegetation divisions refer to Sayre et al. [49].

Latitudinal Range	Total Area (km^2^)	Total Number of Flea Beetle Genera	African Endemic Genera (%)	Flea Beetle Genera Endemic to a Single Latitudinal Range (%)	Number of Vegetation Divisions
40–35° N	219,406	22	0	0	12
35–30° N	1,441,210	23	0	0	13
30–25° N	2,567,593	13	7.69	0	13
25–20° N	2,990,429	12	8.33	0	13
20–15°	3,228,301	32	28.13	0	19
15–10° N	3,620,333	53	47.17	3.77	19
10–5° N	3,673,971	53	47.17	0	18
5–0° N	2,269,300	65	53.85	1.54	19
0–5° S	1,888,226	66	54.55	1.51	27
5–10° S	1,617,538	64	53.12	3.12	27
10–15° S	1,648,111	58	51.72	0	29
15–20° S	1,520,157	44	45.45	0	27
20–25° S	1,201,049	48	47.92	0	27
25–30° S	915,547	51	52.94	3.92	29
30–35° S	478,961	39	43.49	5.13	24

**Table 3:** Metadata for the 15 latitudinal belts that were considered. BIO2: mean diurnal range, BIO3: isothermality, BIO8: mean temperature of the wettest quarter, BIO9: mean temperature of the driest quarter, BIO13: precipitation of the wettest month, BIO14: precipitation of the driest month, BIO15: precipitation seasonality, BIO18: precipitation of the warmest quarter, BIO19: precipitation of the coldest quarter [50].

Latitudinal Range	BIO2 (Mean)	BIO3 (Mean)	BIO8 (Mean)	BIO9 (Mean)	BIO13 (Mean)	BIO14 (Mean)	BIO15 (Mean)	BIO18 (Mean)	BIO19 (Mean)
40–35° N	11.39	38.27	11.01	24.99	72.54	5.323	51.29	38.31	192.3
35–30° N	12.94	40.44	13.78	28.04	25.4	1.265	60.87	9.296	60.28
30–25° N	14.43	43.22	17.55	27.78	4.716	0.06	49.47	1.775	9.149
25–20° N	14.9	46.42	29.45	21.57	6.837	0.122	67.47	10.19	1.752
20–15°	15.11	52.89	31.09	22.97	44.36	0.137	139	29.21	1.894
15–10° N	14.05	59.63	26.5	25.05	192.7	0.814	127.1	88.07	105.8
10–5° N	11.94	68.53	24.58	25.03	223.7	6.895	84.81	195.3	422.1
5–0° N	10.8	78.37	24.64	24.71	197.5	29.09	62.61	295.3	344.7
0–5° S	9.799	78.05	23.63	22.66	215.4	27.19	58.34	403.6	155.5
5–10° S	11.18	69.54	23.43	21.63	213	3.525	83.16	333.9	65.69
10–15° S	12.74	61.17	22.47	18.63	237.1	0.716	104.3	288	7.666
15–20° S	14.16	58.75	24.16	18.39	166.1	2.265	109.2	234.3	15.84
20–25° S	15.27	59.7	24.83	16.41	91.84	2.446	95.73	215.6	11.29
25–30° S	15.54	55.71	22.77	12.61	78.24	5.45	74.59	202.1	22.39
30–35° S	14.41	54.25	18.41	13.32	61.6	13.83	46.66	134.9	70.79

## Data Availability

Upon request, the authors can provide the original data used in this paper.
